# Medical Student and Tutor Perceptions of Video Versus Text in an Interactive Online Virtual Patient for Problem-Based Learning: A Pilot Study

**DOI:** 10.2196/jmir.3922

**Published:** 2015-06-18

**Authors:** Luke A Woodham, Rachel H Ellaway, Jonathan Round, Sophie Vaughan, Terry Poulton, Nabil Zary

**Affiliations:** ^1^ Institute of Medical and Biomedical Education St. George's, University of London London United Kingdom; ^2^ Department of Learning Informatics Management and Ethics Karolinska Institutet Stockholm Sweden; ^3^ Human Sciences Northern Ontario School of Medicine Sudbury, ON Canada; ^4^ St George's University Hospitals NHS Foundation Trust London United Kingdom

**Keywords:** problem-based learning, educational technology, multimedia, Internet, audiovisual aids

## Abstract

**Background:**

The impact of the use of video resources in primarily paper-based problem-based learning (PBL) settings has been widely explored. Although it can provide many benefits, the use of video can also hamper the critical thinking of learners in contexts where learners are developing clinical reasoning. However, the use of video has not been explored in the context of interactive virtual patients for PBL.

**Objective:**

A pilot study was conducted to explore how undergraduate medical students interpreted and evaluated information from video- and text-based materials presented in the context of a branched interactive online virtual patient designed for PBL. The goal was to inform the development and use of virtual patients for PBL and to inform future research in this area.

**Methods:**

An existing virtual patient for PBL was adapted for use in video and provided as an intervention to students in the transition year of the undergraduate medicine course at St George’s, University of London. Survey instruments were used to capture student and PBL tutor experiences and perceptions of the intervention, and a formative review meeting was run with PBL tutors. Descriptive statistics were generated for the structured responses and a thematic analysis was used to identify emergent themes in the unstructured responses.

**Results:**

Analysis of student responses (n=119) and tutor comments (n=18) yielded 8 distinct themes relating to the perceived educational efficacy of information presented in video and text formats in a PBL context. Although some students found some characteristics of the videos beneficial, when asked to express a preference for video or text the majority of those that responded to the question (65%, 65/100) expressed a preference for text. Student responses indicated that the use of video slowed the pace of PBL and impeded students’ ability to review and critically appraise the presented information.

**Conclusions:**

Our findings suggest that text was perceived to be a better source of information than video in virtual patients for PBL. More specifically, the use of video was perceived as beneficial for providing details, visual information, and context where text was unable to do so. However, learner acceptance of text was higher in the context of PBL, particularly when targeting clinical reasoning skills. This pilot study has provided the foundation for further research into the effectiveness of different virtual patient designs for PBL.

## Introduction

Virtual patients are interactive online tools that present learners with simulated patient encounters [1]. They are used in a range of contemporary medical educational settings, including small-group learning, lectures, self-directed learning, and assessment [2,3], as well as in other disciplines such as nursing [4] and primary care [5]. Virtual patients are generally Web-based which allows for a wide range of resources, such as multimedia or multiple-choice questions, to be included in their design.

The use of virtual patients has been linked to the development of learners’ clinical reasoning skills [6] by allowing them to be active participants in a clinical situation, interpreting the available information, and making decisions based on what they know. The design of virtual patients generally follows 1 of 2 models: linear or branching [7]. Branching virtual patients are based on a decision tree that allows learners to make decisions at selected option points, thereby changing their path through the case. In contrast, learner interactions with linear virtual patients do not change the narrative of the scenario. Different paths can have different consequences, which can help learners to develop their clinical reasoning skills in ways that are safe, structured, and rich in feedback and instruction [8].

Research into the effective use of simulation for learning has identified the benefits of feedback and repetitive practice [9-11]. Virtual patients are a form of simulation and many of these factors have been used to guide their design [12]. Low-fidelity simulations, such as virtual patients, have a number of advantages over high-fidelity mannequin-based simulations. For instance, they are cheaper to produce and deploy and can (by being Web-based) be scaled to larger numbers of concurrent users. Indeed, Norman et al [13] argue that there is little educational advantage in using high-fidelity simulations over lower fidelity solutions, whereas Maran and Glavin [14] make a distinction between “engineering fidelity” and “psychological fidelity.” Low-fidelity simulations, such as virtual patients, arguably have a low level of engineering fidelity (ie, the degree to which the physical characteristics of the task are represented) that can reduce their cost without reducing their psychological fidelity (ie, the degree to which skills of the task are captured by the simulation).

St George’s, University of London in the United Kingdom developed a range of virtual patients for use in problem-based learning (PBL) [15]. This was done by adapting existing “paper” cases to include branches where learners could move through a case by selecting different patient-management options [16]. Each option took the learners down a different path, each of which was set up with different consequences for the development of the case. These case designs were rendered in an online virtual patient system so that multiple groups of students could use the same case simultaneously while tracking the different routes they took through the case.

In 2010, St George’s, University of London transformed their PBL curriculum, replacing the paper cases with interactive online virtual patients and delivering this throughout the academic year [17,18]. This initiative was well-received by learners, the majority of whom preferred the virtual patient cases to paper-based cases [16]. This model of PBL has continued to be used to the present day. However, for the technology to be effective and sustainable, it required that the project team take a wider view of how to integrate technology into the PBL learning environment, establishing new procedures and guidelines beyond simply switching the paper resource to a branching virtual patient [17].

The educational community is taking an increasingly holistic view of the role of technology in education, acknowledging that an effective learning exercise depends greatly on the way that is implemented and the context within which it is implemented [19,20]. Ellaway [21] has proposed that virtual patients be considered from an activity-theoretical viewpoint; that learning is not intrinsic within the technological artifacts themselves, and that research should instead focus on the educational activities that virtual patients can be used to mediate. From this perspective, the virtual patient is a part of the scaffolding on which an activity is built along with factors such as the environment in which it takes place and the role of the tutor or facilitator. Educational technologies can be used in a variety of ways by learners with varying degrees of effectiveness [22], and likewise a single virtual patient can be used as a part of many different activities [23].

The need to situate a virtual patient resource within the activities that make use of them is not unique to virtual patients; a similar approach is required to guide the use of any technical artifact used in a learning activity. The primarily Web-based nature of virtual patient resources allows additional forms of media, such as images and video, to be easily incorporated within the virtual patient and their effectiveness should also be considered in the context of the activities in which they are used.

Although there have been several studies that have explored the impact of using video within traditional PBL curricula [24-27], there is little published evidence regarding the impact of using video in PBL that uses branching virtual patients targeting clinical reasoning, particularly for undergraduate learners. Bowen [28] identifies key elements of the clinical reasoning process to be that of data acquisition and the subsequent generation and identification of the problem to be addressed, leading ultimately to the generation of a hypothesis and diagnosis. Studies involving undergraduate medical students demonstrated a reduction in this type of critical thinking in nonbranching PBL following the introduction of video-based cases [25,26]. Kamin et al [29] identified a particular decrease in critical thinking at the point of identifying problems when using video-based PBL (compared to the same information provided as text), attributing this to the learners’ need to perceive and articulate information from video. In contrast, this study also identified beneficial effects attributable to the use of video in other, later, stages of critical thinking. Studies focusing on postgraduate learners have identified similar benefits to the use of video [24]. De Leng et al [27] explored learner perceptions of video in a traditional PBL setting and proposed a series of guidelines for its effective use. They identified 4 key areas in which videos could enhance and add value to PBL cases: they were more authentic and illustrative, they provided a more comprehensive view of a scenario, they were more challenging for the learner, and they were more memorable. However, these guidelines did not take into account the particular characteristics that branching virtual patient resources brought to these activities, nor was there a specific focus on the development of clinical reasoning skills. Therefore, there is a need for evidence-based guidance on how to use video when developing Web-based virtual patients for use in PBL activities.

Our starting hypothesis was that virtual patients, and particularly branching virtual patients, are better suited to developing clinical reasoning skills than traditional PBL [15,30,31] and that (based on evidence from previous studies) the introduction of video elements to PBL can reduce the ability of undergraduate students to engage in critical thinking [25,26,29]. More specifically, we wanted to explore whether the use of video within a branching virtual patient for PBL could reduce the development of students’ clinical reasoning skills due to the difficulty of critically evaluating the information provided in a video format compared to a text format. Therefore, we designed the study to address the question of how undergraduate medical students interpret and evaluate information provided by video, when compared with text, presented in the context of a branched interactive online virtual patient designed for PBL?

## Methods

### Overview

We created an educational intervention in the form of an adapted virtual patient case, in which the early stages of the case replaced text content with video. This virtual patient case was introduced into one week of the PBL curriculum for undergraduate medical students at St George’s, University of London.

**Figure 1:**
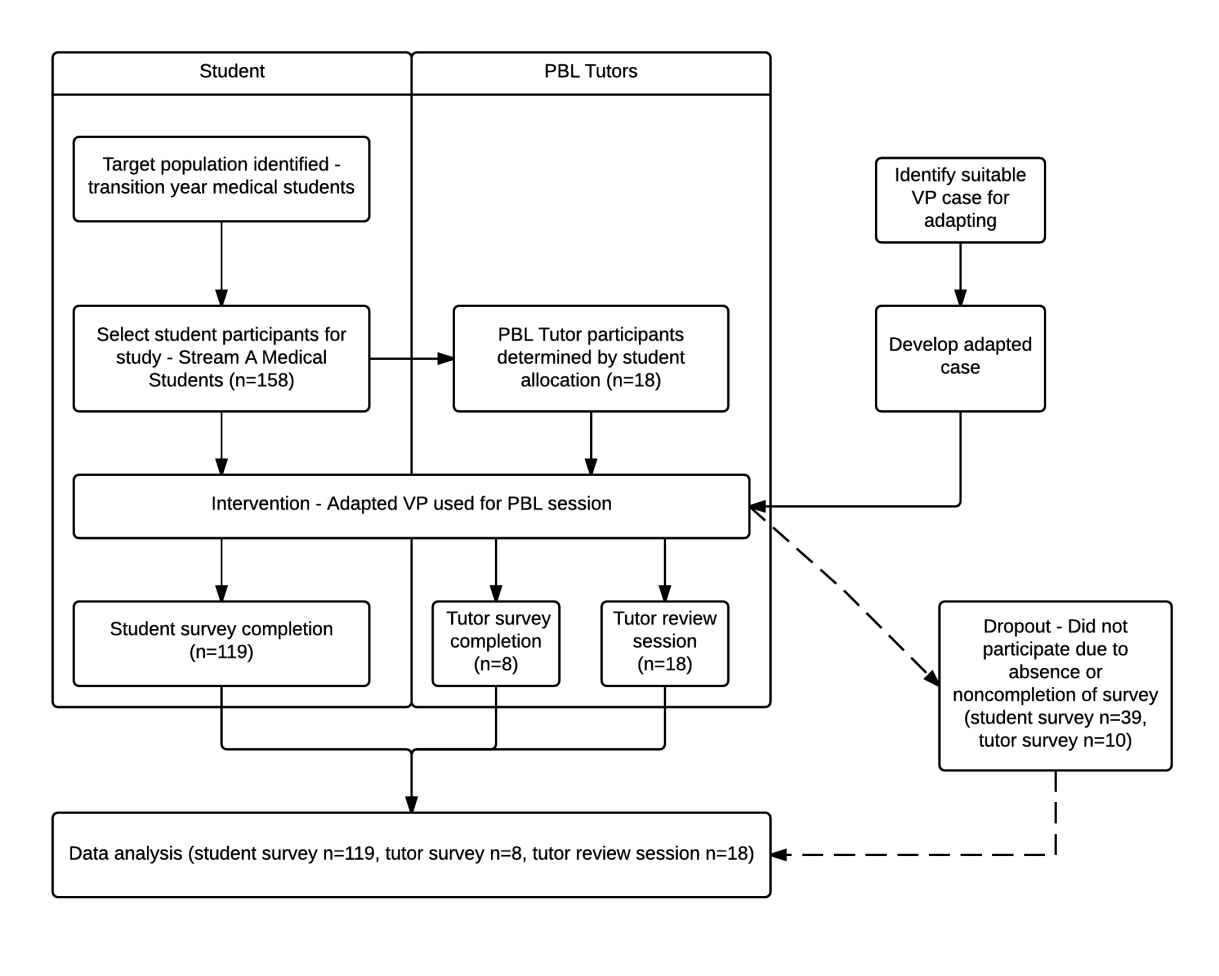
Flow diagram describing study design for using virtual patients (VPs) in problem-based learning (PBL).

We used a mixed-methods approach [32,33] to capture the experiences of these students and the PBL tutors that facilitated the sessions. All participants had previous experience of PBL tutorials that used text-based virtual patients, so text was used as a baseline for comparison with video. Utilizing a convergent parallel study design [33] as described in Figure 1, we surveyed students and tutors through written questionnaires and ran a review session with tutors to capture their verbal feedback and merged the results in the analysis phase.

### Participants and Setting

The primary participants for the study were undergraduate medical students enrolled in the transition year at St George’s, University of London. This was the second year of the program for graduate entry students and the third year for undergraduate entry students and was the point at which these 2 program streams joined.

Students undertook a program of PBL tutorials during the transition year. Running twice a week, there were 18 themed tutorials, each of which took place over 3 separate timetabled 3-hour sessions. For practical reasons, a student cohort was divided into 2 streams, with the groups completing the PBL tutorials at different stages of the year. Students from one stream (stream A) received the intervention in October 2013. Because PBL sessions were a mandatory component of the course, all students in the selected stream participated in the learning activity. Participation in the feedback activity was encouraged, but was not mandatory. All survey responses were anonymous.

Each PBL group consisted of 8 or 9 students, a mix of graduate entry and undergraduate entry learners. The PBL sessions were facilitated by tutors who also participated in the study. Their role was noninterventional; they did not teach but guided the session to ensure that the groups stay focused and covered the requisite learning objectives. Each PBL group was facilitated by a tutor who worked with the same learner groups throughout the year. The role of a tutor in PBL requires a specific approach and a particular set of guidelines must be followed; all PBL tutors received training in their roles and PBL techniques, but were not selected on the basis of any subject knowledge. The tutorial in which the intervention was introduced was conducted in the same manner as any other PBL session; experienced PBL tutors did not require or receive any specific training related to the intervention. However, they were informed before the date that the intervention would be introduced and given the opportunity to raise questions about the study with the research team.

The study was reviewed and approved by the chair of the St George’s, University of London Ethics Review Board and approved by the undergraduate program course director. To provide assurance that students received no advantage through either participation or nonparticipation, all students were provided with access to both the video and text-based versions of the tutorial after the PBL session had taken place using the institutional learning management system. The video virtual patient was reviewed by the module leads before the intervention was delivered to confirm that the content was suitable for use, accurate, and that all the required information for the case was still delivered. The module leads agreed that the content was suitable and gave their consent to its use.

### Intervention

One section of a preexisting virtual patient PBL case was adapted by replacing the textual information with video clips. We used the following predetermined criteria to select a suitable virtual patient case for adaptation from the existing St George’s, University of London PBL curriculum:

1. The case had to have a narrative that could be staged using the equipment and performers available in the St George’s Advanced Patient Simulator simulation and skills training center. This eliminated cases that took place primarily in nonclinical environments.
2. The part of the case to be filmed also had to require learners to interpret only information that could be effectively shown and visualized through video, meaning sections of the case which required analysis of detailed test results, scans, or other similar information were not considered suitable for video representation.
3. The timing of the case in the curriculum was also a critical factor; it was necessary that it took place early in the year so that students were not so familiar with text-based cases that it would prejudice their perceptions against a change to video.

Having reviewed all the available virtual patient cases against these criteria to determine their suitability for adaptation, we selected the second part of a 3-part virtual patient case regarding a patient suffering an abdominal aortic aneurysm. Considering the established typology for virtual patients [7], the selected virtual patient had a number of defining characteristics. The case was presented in the English language using a branching path model to target undergraduate medical students in the context of a PBL tutorial. It was designed to be used in a PBL tutorial with standard-sized groups of 8 or 9 participants and a duration of 3 hours. In the virtual patient scenario, students were asked to assume the role of a Foundation Year 2 (second year of postgraduate training) doctor, with the focus being decisions to be about patient treatment [17].

We storyboarded the first 9 stages of this for filming, taking in the first 2 decision points that learners were required to negotiate during the PBL session. This was the first case that was scheduled to run during the academic year, helping to ensure that the learners were not already too familiar with the text-based tutorials used elsewhere in the curriculum in advance. Each clip was designed to provide both the scenario narrative and the relevant information that learners needed to make effective patient-management decisions.

The video material was created in partnership with the St George’s Advanced Patient Simulator center because they had the facilities to create an approximation of the required settings. The videos were recorded in simulation rooms from 4 angles, with sound captured from room-mounted and individual wireless microphones worn by performers.

**Figure 2:**
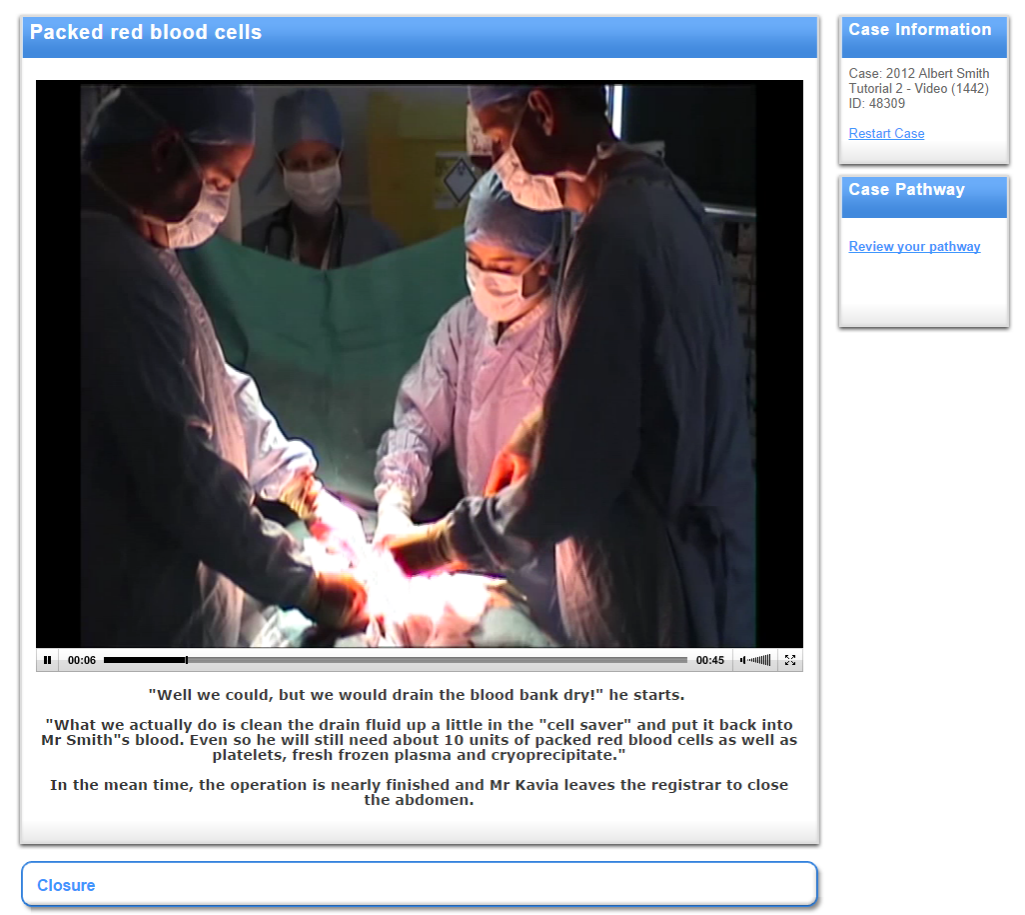
Screenshot showing a video clip embedded in the online virtual patient activity used in this study.

The filming was completed over 2 half-day sessions using volunteer actors and 5 cameras. Four cameras were fixed-viewpoint cameras available in the simulation center, whereas an additional portable camera was used to capture close-up shots and other viewpoints. The simulation center was used to stage scenes representing a recovery room, an operating theater, and a ward environment. Approximately 4 hours of footage was captured from each camera and this was edited down into 9 video clips varying from between 45 seconds to 4 minutes in length. These video clips were then embedded into the virtual patient case, replacing the text in this part of the tutorial. Examples of the video clips are provided in Multimedia Appendices 1 and 2. The video content was reviewed for suitability and accuracy by the academic leads for the relevant module of the course and approved for use. A screenshot of a video in a virtual patient case is shown in Figure 2.

Teaching sessions took place in dedicated small-group teaching rooms; students and tutors were arranged around a table with an Internet-connected computer workstation attached to an interactive SMART Board [34] and projector situated at one end of the room, which was used to display the virtual patient case to the group.

### Instrument Development

We conducted this mixed-methods study using structured and unstructured data gathered from 3 sources: a student survey, a survey of PBL tutors, and a discussion and review session with PBL tutors.

We developed the survey instrument from an established instrument for exploring student experiences using virtual patients [35-37]. Questions were added regarding students’ perceived ability to evaluate the information available in the scenario, their understanding of the context provided by the scenario, and their sense of engagement with the case. There were 19 questions in total. The first 2 questions (room number and course type—undergraduate entry or graduate entry) were used to categorize the data for analysis and the third question asked for a perception of how many times their group watched each video on average. The remaining questions provided for structured responses in the form of a multiple-choice or Likert scale answer followed by an unstructured response to provide further detail or explanation. The full survey instrument is provided in Multimedia Appendix 3. Given the established basis of the parent instrument and time constraints in executing the study, we did not pilot the survey instrument further. We provided the survey instrument to student participants in paper form as part of the packs that accompanied the PBL session to encourage on-the-spot completion. We entered the paper-based responses into a Web-based system [38] to allow for combined reporting and analysis.

The experiences and thoughts of the PBL tutors were captured through a review session and a distinct survey instrument tailored to the tutor experience. The survey instrument was based on those developed in a previous study relating to tutor perceptions of virtual patients in a virtual world [39] and adapted to the context of this study. The survey instrument (see Multimedia Appendix 4) was provided to the tutor participants in paper form for on-the-spot completion at the time of the PBL session in which the intervention was delivered. One of the researchers (LW) conducted the review session in November 2013 and a semistructured approach was identified as being appropriate for the review session due to the research team’s existing knowledge of the domain [40] and a question script was developed to guide the discussion (see Multimedia Appendix 5). The session was audiorecorded and later transcribed for analysis. The participants in the review session were PBL tutors that had facilitated the tutorial in which the video-based virtual patient intervention was introduced.

### Analytic Approach

We analyzed the unstructured free-text data from the survey responses and reviewed session transcripts using a theoretical thematic analysis approach [41]. The data were manually coded by one of the authors (LW) using ATLAS.ti software [42] and the codes generated were developed through iterative readings of the datasets. Individual sentences in transcripts and free-text responses were identified as the units of analysis for coding [43] to ensure that all themes expressed could be identified with sufficient granularity. We used an open-coding approach for the first reading of the data, in which themes grounded in the data were noted. A second reading continued with this approach, identifying information overlooked during the first iteration. In accordance with a theoretical thematic analysis model and in contrast to an inductive analysis of the data, the coding process was conducted with a view to the specific area of research examined in this study and did not attempt to codify the responses beyond this context. Subsequent iterations moved toward an axial coding model [44]. With each reading the generated codes were further refined, thematic linkages between codes were noted, and codes with common meaning were merged and grouped. After the sixth reading of the data, all the thematic groupings were clearly distinct and no new codes emerged, and the researchers were satisfied that the coding process had allowed a number of broad, descriptive themes to be identified in the data.

We analyzed the structured responses by converting Likert scale matrix values to ordinal form (strongly disagree=1; strongly agree=5) and by generating descriptive statistics. We categorized data by course of study (undergraduate entry or graduate entry) to control for any bias caused by differing levels of experience among student participants and we used 2-tailed Mann-Whitney *U* tests to identify if there were any statistically significant differences between the 2 groups.

## Results

### Overview

Out of 158 students registered to attend the PBL session, 119 responded to the student survey giving a response rate of 75.3%, although some students did not answer every question. We considered this response rate to be satisfactory and attributed noncompletion of the survey to a combination of absence from the session or students electing not to provide feedback. In addition, responses were not received from 2 of the 18 student groups, suggesting that the tutors responsible for collecting the student responses in those rooms had either not distributed or simply did not return the survey instruments. In total, students provided 274 open-text comments in their survey responses.

The tutor survey received 8 responses, a response rate of 44% (8/18), with 21 open-text comments. In both datasets, responses that were null, “n/a,” or simply “no” when asked for further details were excluded. Due to the small sample size (n=8) for the PBL tutor survey response, we determined that generating descriptive statistics for this would be of little value and unreliable, although the unstructured tutor responses were included in the qualitative analysis. The low response rate for the PBL tutor survey was attributed to the timing of the survey data collection because it took place at the end of the session when tutors had a number of other tasks to complete, such as gathering materials and feedback from students, meaning that time for them to complete the survey was scarce. It was also noted that the tutor survey had been provided in the information pack provided to tutors, which also included the student survey forms. The 2 instruments were both printed on white paper and were, therefore, not immediately distinguishable. Although tutors had been previously briefed, a possible explanation for the low response rate was that they were unaware the tutor survey had been included.

Of the 16 groups that returned responses, 7 reached a consensus on the number of times each video was watched, 6 stated that they had watched each video once on average and 1 group reported having watched each video twice. No consensus was reached for the other 9 groups, with responses ranging between 1 and 2 viewings.

The descriptive statistics for the Likert scale items from the student participants are shown in Table 1.

**Table 1:** Descriptive statistics for Likert scale student survey responses (N=119).

Statement and medium encountered		Responses, n	Mean score (SD)
**While working on this case, I felt I had to make the same decisions a doctor would in real life.**			
	Text	119	3.87 (0.75)
	Video	116	3.82 (0.90)
**While working on this case, I felt I were the doctor caring for this patient.**			
	Text	119	3.53 (0.85)
	Video	114	3.41 (1.04)
Watching the scenario take place in the videos made me feel more emotionally involved with the case than when playing the role of an F2 doctor in the text.		118	2.99 (1.06)
Playing the role of an F2 doctor in the text-based parts of the tutorials increased my engagement with the scenario compared with watching the videos.		119	3.30 (0.88)
The use of video brought the scenario to life.		119	3.49 (1.02)
The use of video made the scenario more memorable.		118	3.62 (1.07)
The use of video influenced the option choices that my group made.		119	2.94 (0.87)
The use of video helped me to relate the scenario to real-life experience.		119	3.49 (0.93)
I was able to obtain all the information from the videos that I needed in order to make informed patient-management decisions.		119	2.99 (1.03)
I felt that it was easier to identify relevant information from text than the videos.		119	3.75 (1.00)
The use of video had a positive impact on the group discussion.		114	3.29 (0.89)

To control the impact of PBL groups with students with different levels of prior experience we categorized data by group; graduate entry students and undergraduate entry students. This yielded 2 independent ordinal datasets for each Likert item. We ran nonparametric 2-tailed Mann-Whitney *U* tests on the datasets to test whether the distributions for graduate entry and undergraduate entry students were significantly different at the 5% level (*P*<.05) (see Table 2). In each case, the null hypothesis (ie, that the 2 groups had the same distribution) could not be rejected indicating that the graduate entry or undergraduate entry status of students, and thus the different nature of the prior experience of these 2 groups, did not significantly impact on their experiences of the intervention and did not have a material impact in skewing the data. We concluded that responses for the different groups did not have to be separated in the analysis and that conclusions drawn would be applicable and generalizable across both groups.

**Table 2:** Descriptive statistics for Likert survey items categorized by learner stream (undergraduate entry or graduate entry).

Survey question		Graduate entry (n=46)			Undergraduate entry (n=73)			*U*	*P*
		Responses, n (%)	Mean (SD)	SEM	Responses, n (%)	Mean (SD)	SEM		
**While working on this case, I felt I had to make the same decisions a doctor would in real life.**									
	Text	46 (100)	3.87 (0.72)	0.11	73 (100)	3.86 (0.77)	0.09	1651.50	.87
	Video	46 (100)	3.96 (0.89)	0.13	70 (96)	3.73 (0.90)	0.11	1341.50	.10
**While working on this case, I felt I were the doctor caring for this patient.**									
	Text	46 (100)	3.35 (0.97)	0.14	73 (100)	3.64 (0.75)	0.09	1451.50	.18
	Video	45 (98)	3.38 (1.09)	0.16	69 (95)	3.43 (1.01)	0.12	1533.50	.91
Watching the scenario take place in the videos made me feel more emotionally involved with the case than when playing the role of an F2 doctor in the text.		46 (100)	3.07 (1.08)	0.16	72 (99)	2.94 (1.04)	0.12	1545.50	.52
Playing the role of an F2 doctor in the text-based parts of the tutorials increased my engagement with the scenario compared with watching the videos.		46 (100)	3.24 (0.87)	0.13	73 (100)	3.34 (0.88)	0.10	1581.00	.57
The use of video brought the scenario to life.		46 (100)	3.48 (0.91)	0.13	73 (100)	3.49 (1.09)	0.13	1601.50	.65
The use of video made the scenario more memorable.		46 (100)	3.63 (1.08)	0.16	72 (99)	3.61 (1.07)	0.13	1633.00	.89
The use of video influenced the option choices that my group made.		46 (100)	2.91 (0.81)	0.12	73 (100)	2.96 (0.90)	0.11	1670.00	.96
The use of video helped me to relate the scenario to real-life experience.		46 (100)	3.52 (0.94)	0.14	73 (100)	3.47 (0.93)	0.11	1589.50	.59
I was able to obtain all the information from the videos that I needed in order to make informed patient-management decisions.		46 (100)	3.17 (0.90)	0.13	73 (100)	2.88 (1.09)	0.13	1424.00	.15
I felt that it was easier to identify relevant information from text than the videos.		46 (100)	3.67 (0.94)	0.14	73 (100)	3.79 (1.04)	0.12	1514.00	.33
The use of video had a positive impact on the group discussion.		43 (100)	3.40 (0.88)	0.13	71 (97)	3.23 (0.90)	0.11	1394.00	.41

### Analysis

Our thematic coding process drew on the unstructured responses of both tutors and students, and led to 67 distinct codes grounded in the data during the open-coding process. Five codes were automatically generated using ATLAS.ti [42] for the purpose of categorizing the open-text survey responses and did not relate directly to the research question. Therefore, we disregarded these for the purpose of the thematic mapping exercise.

Table 3 shows the high-level themes we identified in the analysis. We identified thematic links between the remaining codes by reviewing the codes for similarities in meaning and relevance. As a result of this exercise, 8 clear thematic groupings emerged (the level of engagement appeared twice with some identifying text as more engaging and others feeling that video was more engaging).

**Table 3:** Summary of high-level themes identified and the number of quotations coded against each theme.

High-level theme	Code-quotation count
Video made the scenarios more real	70
Hard to identify key information in video	68
Video more engaging	55
Poor sound quality	44
Text can be reviewed	34
Would favor a text script to complement video	31
Text more engaging	21
Video slows the pace of PBL	15
Video well-suited to showing procedures	10

#### Level of Engagement

We found quite varied perspectives on the effectiveness and desirability of using video as part of the PBL process. For instance, 55 comments indicated that the video had had a positive impact, whereas 20 statements described a negative effect. Those students who described a positive engagement with video noted that it provided the scenario with more “immediacy” and “involvement.” Several students raised the countervailing idea that text “allows more room for imagination.”

The structured data reflected a similar lack of consensus when considering the merits of using video to heighten engagement. We noted before the intervention that the role of the student was altered by the introduction of video; when using text the student was addressed as if they were the doctor, whereas in the video the student was at most observing the doctor. We anticipated that this may have had a negative impact on student engagement with the scenario, although there were other more positive factors, such as providing richer visual information and context. To explore this effect in more detail, individual students were asked whether they felt they had to make the same decisions as a doctor and whether they felt they were the doctor caring for the patient for both the video and text components of the virtual patient. We used sign tests (*Z*) because of the ordinal and dependent datasets to test whether the median values were different for video and text (Table 4). In each case, the null hypothesis (ie, that the distributions were the same) could not be rejected at the 5% level (*P*<.05), indicating that the use of video did not significantly impact on student responses to this statement when compared to their response for text.

**Table 4:** Results from 2-tailed sign test (*Z*) for individual student responses to Likert items comparing text and video.

Likert item	Negative differences, n	Positive differences, n	Ties, n	Total, n	*Z*	*P*
While working on this case, I felt I had to make the same decisions a doctor would in real life.	23	23	70	116	<0.001	>.99
While working on this case, I felt I were the doctor caring for this patient.	27	25	62	114	–0.139	.89

#### It Can Be Harder to Identify Relevant Information From Video

Students found that it was harder to identify relevant information in the video compared to text. Many of the students identified that they missed key bits of information in the video and that this confused the group. One participant commented that the video was “unclear and lacked direction and confused us more as a group.” This perceived information deficit was felt by some participants to have reduced the quality of discussion and that they “learned more and had more information available to discuss with text in front of me.”

#### Text Can Be Reviewed More Easily Than Video

We identified that a key advantage of text was that it can be reviewed and revisited more easily and on an individual basis by students during the PBL session. Many students wanted to be able to refer back to the text and identified that the nature of video meant that during discussions they did not have the ability to refer back to the source material. Although the video could be replayed, and many groups confirmed that they played the video multiple times, it was not possible to view the video and discuss simultaneously. One student commented that “it’s easier to check facts when debating” using text.

#### Video Slows the Pacing of Problem-Based Learning Activities

Many of the students and tutors identified that the use of video had a significant impact on the pace of a PBL session. Some students identified that this caused an increase in the overall length of the session by approximately 30 minutes. One tutor in the review session identified that the pace and responsiveness of the discussion was slowed by the introduction of video:

I think we took longer because after they watched the video we still had to stop and talk about things that the video raised, but we couldn’t talk about them at the time as we were watching the video, so for us the case actually took longer than it might have done if we were to raise learning objectives and discussing.

The student survey indicated that 38.5% (45/117) of the students felt that they on average watched each video twice or more than twice, which also had the effect of slowing the pace of the session. Comments also considered this in relation to the greater difficulty in reviewing video compared to text, stating that they had to “repeatedly watch” the video. It was suggested that providing a text transcript would alleviate this issue by eliminating the need for repeated viewings.

#### Video Made the Scenario Seem More Real

Many students commented on ways in which they felt the use of video made the scenario more real. The comments reflected a broad range of reasons, but with a common thread that the use of video “brought it to life.” Some comments related to the students being able to identify themselves and their role in the scenario: “it made it more real as if I was present whilst the whole situation was happening.” Another felt that video “portrayed the urgency of the decision that needed to be made.”

A particularly common observation among the students was that the video provided additional visual cues that were not present in the text. The nature of these visual cues varied greatly, some students identified the impact of seeing the social interactions taking place or of seeing reactions and facial expressions in the video. Others mentioned the importance of observing the environment in which the patient encounter occurred, saying that it “contextualizes the scenario” and provided “clues about what is in environment, IV drips, blood, etc.” Additionally, some students described the impact that these visual clues had on the decision-making process and stated that “visualizing blood loss on the video altered our initial opinion on what to do next.”

#### Video is Well-Suited to Displaying Procedures

A number of the participants (both tutor and student) suggested that video could provide particular advantages over text in representing clinical procedures. A significant component of the video we used showed a simulated surgical procedure to treat an abdominal aortic aneurysm. However, we should be clear that the clip was not intended to teach the procedure, but to further the narrative of the scenario. Nevertheless, its inclusion triggered responses indicating that video was perceived by students to be a superior way of learning about such procedures compared to text, comments including “some of the practical procedures (ie surgery) are better explained and understood if it was demonstrated ‘in action’ in the form of a video.” Other comments recognized the benefits of seeing video of procedures, but questioned the value of embedding them within patient scenarios, stating that it would be just as valuable to link to YouTube videos showing the same procedures.

#### Students Favor a Combination of Text and Video

**Figure 3:**
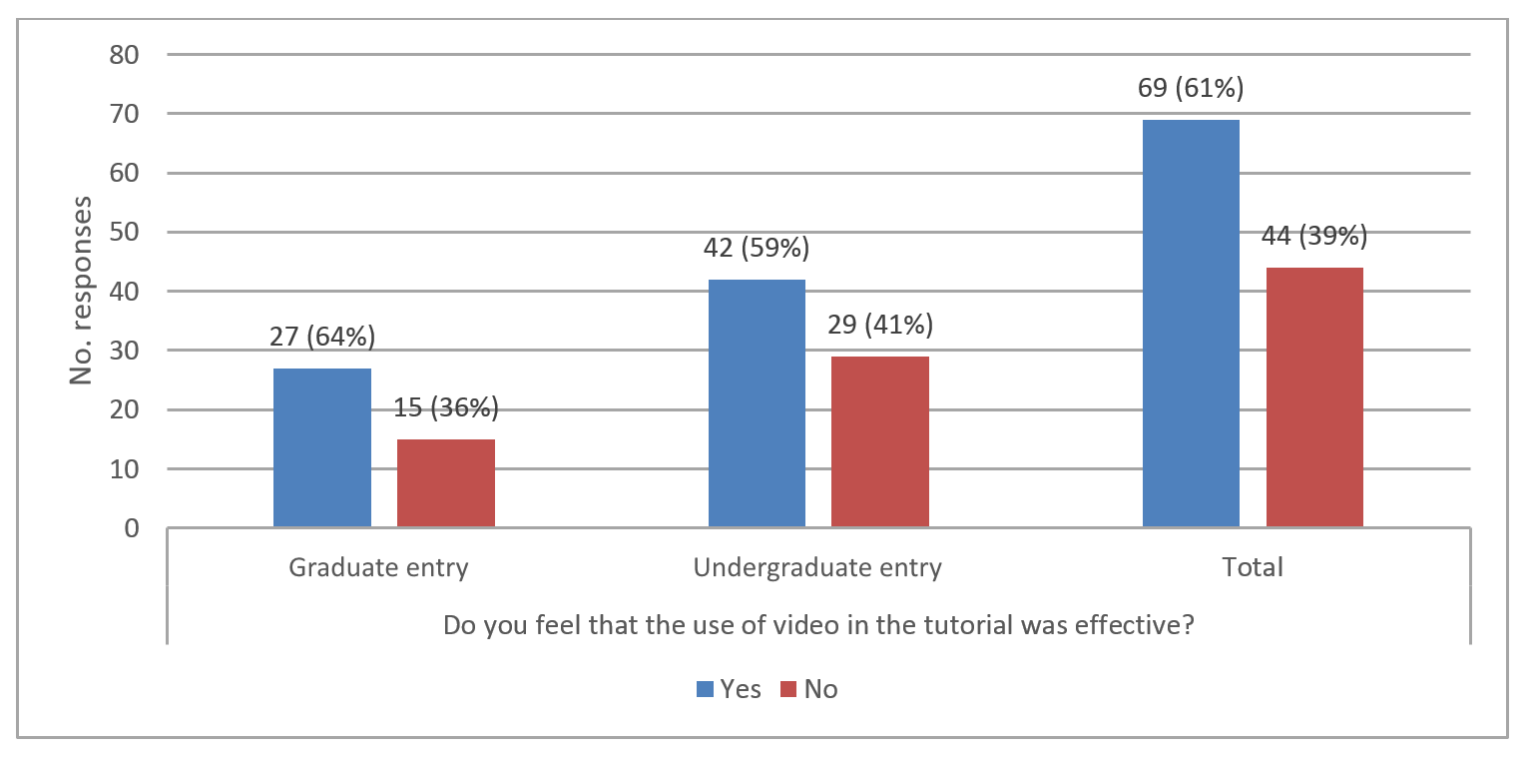
Bar graph of student responses to question “Do you feel that the use of video in the tutorial was effective?”.

**Figure 4:**
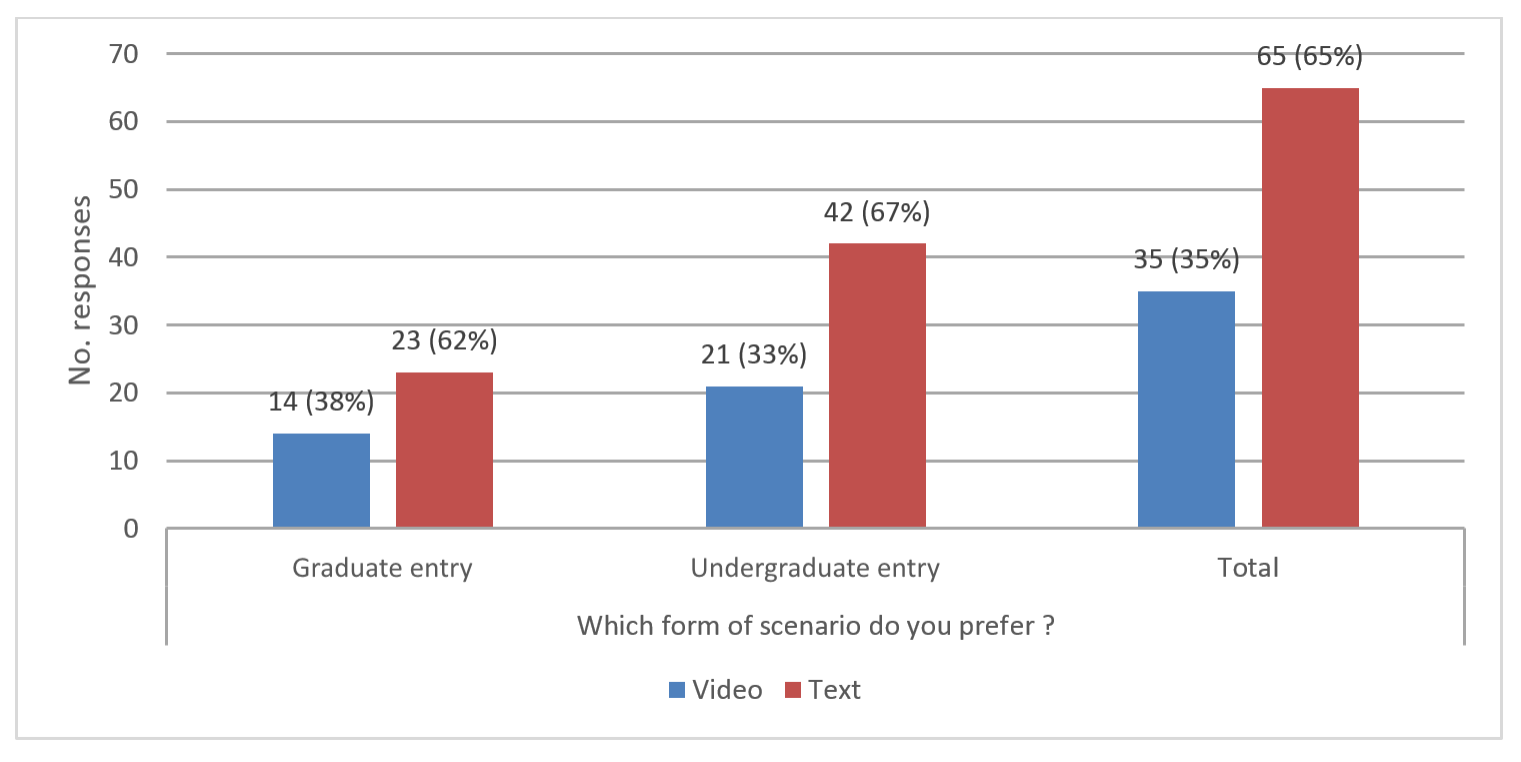
Bar graph of student responses to question “Which form of scenario do you prefer?”.

We asked student participants whether they felt that the video was effective and whether they preferred video or text. The responses to these questions showed similar patterns regardless of year of study, with a majority of students feeling that the use of video was effective (Figure 3). However, when asked to state a preference for video- or text-based scenarios, the majority of students expressed a preference for text (Figure 4).

Several students suggested that an optimum arrangement would be to have a combination of video and text; one student suggested “a mix so memorable but also easy to understand.” Another agreed, commenting that a combined approach “would allow us to see the key case easily whilst seeing a more realistic scenario in the video.” Others asked for a text transcript of the content in the video. Other views indicated that the combination approach would best be achieved by providing the video first then the text equivalent.

#### The Quality of the Video Resource

Many tutors and students commented on the quality of the video resource and the impact that it had on the session. A particular concern of many was the perceived poor sound quality, with participants stating that the sound was “hard to hear,” “muffled,” and “occasionally distorted.” Examination of the video clips revealed that there was audible distortion present in the original recording at 2 points in the videos. Inspection of the rooms in which the sessions took place also revealed that the character of the frequency response of the audio systems in those rooms had the effect of muffling the speech in the clips and accentuating the background noise making the speech less clear than had been apparent when preparing the clips using headphones.

Additional comments were made about the quality of the video relating to the editing of the clips (eg, cuts were too frequent) and, in particular, the quality of the acting. The actors in the clips were volunteers from the project team and as a consequence were perceived to lack proficiency. It was felt by some that the acting quality distracted from the learning task and that “random clips of poor acting doesn’t translate information effectively.”

## Discussion

### Principal Results

Our study investigated the impact of video clips replacing related text content on a PBL session run using an interactive virtual patient. In particular, we considered the ability of students to process and evaluate the information provided in the video. What became clear on analysis of the results was, although a number of themes emerged, the findings showed a diversity of opinions among students. When asked the question explicitly, a majority of the student participants indicated that they preferred text-based virtual patients for PBL, while acknowledging the benefits of using video in certain circumstances. The contrast between the majority of students’ stated preference for text, yet their widespread identification of the benefits of video, may potentially be explained by understanding the motivation and challenges faced by today’s medical students, who are required to assimilate a significantly increased volume of knowledge and to continue to do so throughout their professional development without a comparable increase in time [45]. Sobral [46] described the motivation of medical students as depending on both extrinsic and controlled factors (ie, the course structure and need to pass certain assessment targets) as well as intrinsic and autonomous ones (ie, the enjoyment of learning). Our results indicate that extrinsic factors were of primary importance to our students, who were necessarily focused on acquiring the ever-widening pool of knowledge needed to qualify and pass their exams. The introduction of video required learners to employ a greater level of critical analysis to extract and evaluate the available information; hence, the students expressed a widespread preference for text.

Participants recognized the value that video provided in terms of engagement and the provision of visual information, commenting that it brought the scenario to life for them. However, the additional challenges that video brought were also demonstrated clearly in our results; the widespread belief among participants was that information was harder to identify in a video clip. Existing studies identify that the use of video makes the initial stages of critical thinking more challenging for undergraduate students [25,26,29], particularly at the problem-identification stage that requires the information provided to be evaluated and synthesized by students. This is made more difficult using video because students have to filter and evaluate a larger volume of information, including visual and auditory information, to extract the key points. Cognitive load theory describes the nature of this increased challenge; learning is impaired when the cognitive load of a task is greater than an individual’s working memory [47]. The introduction of video imposes cognitive load that learners perceive as extraneous and unnecessary for them to achieve their immediate learning objective, which is to acquire the knowledge to pass exams. It is arguable that because the goal of the branching virtual patient is to develop clinical reasoning skills for real-life practice, the ability to critically filter the available information is far from extraneous. However, as a consequence of this potential misalignment in the perceived learning objectives for the virtual patient between educators and students, the effect was that video was perceived to be less efficient than text as a method of acquiring information. Further research is required to investigate this contention and to more fully understand learner motivation when participating in PBL activities.

The time taken during the learning activity was shown to be a priority issue among participants, with many responses noting that video slowed the pace of the PBL session. Groups were generally unable to agree on the extent of this effect, however, with only a minority of groups able to reach a consensus on how many viewings of each video were required. Moreover, video was considered harder to review multiple times to identify the information; repeat viewings had to always be watched by the whole group because an individual could not rewatch the video in isolation. Text, on the other hand, provided scrolling was not necessary, allowed all the information to be visible on screen at one time and could be reviewed independently by individuals without requiring the whole group’s attention. This loss of individual agency over the resource when wanting to review the information compounded the difficulty that learners faced in effectively evaluating the information provided and was a key factor in student perception of its effectiveness. This supports the conclusions reached in previous studies [48], which identified the importance of student control when using multimedia resources.

Student perceptions of the technical qualities of the video also impacted upon the perceived effectiveness of the resource. In videos where the students perceived the audio quality or the proficiency of the actors in the clip to be deficient, many commented that it had a negative effect on their learning. The key point from this is that video is not intrinsically educationally useful (or not) because production quality, editing, and a number of other contextual factors can separately impact its utility.

Existing work has proposed specific design principles for virtual patients [12], which include the appropriate use of media and the authenticity of the interface. Although the proposed principles do not address virtual patients for PBL specifically (which have very specific requirements and warrant consideration in isolation from virtual patients intended for other purposes), the use of video as media within a virtual patient for PBL has a profound impact on the authenticity of the virtual patient interface as a means for engaging the student with the scenario. The principles identify that the use of media should be preferred when it provides a superior means for explaining or providing information to learners. Comparing our results with the factors identified by De Leng et al [27], learners acknowledged that video can provide a comprehensive and illustrative representation of a scenario, it can convey a large amount of visual information, and it can help to make the scenario feel more real. Our study did not attempt to establish whether the information provided in video is more memorable and further research would be required to address that particular question.

However, the claim that video is more authentic is potentially a contentious one. Differing perspectives on what is represented by authenticity make it difficult to validate claims of video being more authentic; the concept of “thick” authenticity suggested by Shaffer and Resnick [49] identifies different types of authenticity in a learning experience, each of which is interdependent with other types. Similarly, the literature on simulation distinguishes between engineering fidelity and psychological fidelity [13,14]. Several of the participants in this study felt that the use of the video made the scenario seem more real, suggesting increased authenticity, and it is clear that the means in which video represents a scenario mirrors real life more closely than text (ie, video provides a higher level of engineering fidelity). Yet when viewing the intervention as a learning activity in the context of the PBL session itself as proposed by Ellaway [21], we must also consider other factors: the video clips were paused and watched multiple times and decisions were reached by consensus without the pressure and time restrictions that would be present in real life. This indicates that the use of video did not serve to increase the psychological fidelity of the learning activity. Given this wider context, the suggestion that the learning activity is more authentic when using video than it is when using text begins to break down and raises the further question of whether authenticity should necessarily be an aspirational characteristic for a learning activity. We have established that the increased challenge provided by video during this intervention reduced the efficiency in which learners were able to achieve their learning goals. Norman et al [13] report that the fidelity of a simulation has little bearing on the effective transfer of learning and our results suggest that indiscriminate use of video aiming to simply increase the authenticity of virtual patient resources may show a similar pattern, particularly if little thought is given to the authenticity of the learning activity mediated by the virtual patient.

However, if effectively targeted to information where it is well-suited, our results indicate that the use of video can be an effective complement to text in PBL activities. It was widely commented in our study that video was well-suited to demonstrating procedures in a way that text cannot. A combined approach, favored by many of the participants, in which a combination of text and video is used, would provide a means to focus the use of video on areas of the virtual patient where it was beneficial to the intended learning activity, while using text to efficiently deliver learning in the areas where it is most effective. The provision of a text transcript that accompanies the video would run counter to the principles proposed by Mayer’s cognitive theory of multimedia learning [50], who points to a redundancy effect achieved when utilizing multiple modalities of delivering information in multimedia resources [51]. However, it is noted that this study is primarily focused on individual and self-directed learning materials. Our results indicate that the group dynamic in PBL sessions based around branched virtual patients may warrant an alternative approach.

### Limitations

This study represents a first step toward investigating the effects of such interventions, piloting the use of video within an interactive virtual patient designed for PBL. However, there are a number of limitations to the study:

1. The findings from this study are based on a single intervention and setting. Further research with a greater number of similar interventions will be required to validate the findings and to establish the broader generalizability.
2. Although the student survey instrument was based on an existing validated instrument [35,36], both this and the tutor survey instrument could have been more robustly validated. Our decision not to engage in substantial validation reflects the pilot nature of the study.
3. Our focus on experience and perception rather than quantifiable outcome measures (ie, exam or clinical skill performance) reflects the broad and relatively unstructured nature of PBL outcomes [52] and the challenges of assessing PBL as a whole [53]. For a pilot study, we were more interested in understanding how learners responded to these different stimuli and how they understood and rationalized these responses rather than quantifying their responses.
4. Although the use of self-reported data would be a potential limitation for a more quantitative study (because of recall and response bias), this was not a significant concern given the proximity of survey completion to the educational event, the use of multiple sources of data, and the pilot nature of the study.
5. Although several students identified that videos of procedures would be beneficial and provide value, the piloted intervention did not include instructional material targeting knowledge of procedures, instead including a simulated procedure to serve the narrative of the scenario. Therefore, the suggestion that video may be well-suited to targeting this area has not been tested in this study and should be the focus of future inquiry.
6. There was widespread dissatisfaction with the quality of the sound in the video clips that were developed for the intervention and this seemed to have had a substantial impact on learner perceptions of the utility of the resource. There was some suggestion that the sound on the clips was not clearly audible using the equipment in the PBL rooms and that this may have exacerbated some of the effects we observed. Learners played clips multiple times to fully understand the provided information may have slowed the PBL process more than it otherwise would have done. Any future studies should include procedures to test the media in the PBL rooms as well as on individual workstations to ensure that it plays with sufficient clarity in that environment.
7. We felt that learner perceptions of the number of times they had played video clips was a relatively crude and unreliable measure and that in future work we would seek to use log data to track the number of times a clip had been played.
8. Finally, this study examined the effect of the intervention at a specific institution, where all the learner participants in the study were previously familiar with the PBL process and had experience of text-based scenarios as a baseline experience. More research is required before the conclusions reached here can be safely generalized to other educational settings. We also urge caution in generalizing our findings to other types of learning activity in which learning may be self-directed or lecture-based. Indeed, our finding that the circumstances of the PBL activity reconfigured the utility of the video and text resources would suggest that the utility and efficacy of video material should be explicitly tested for different educational activities and settings.

### Conclusions

This pilot study introduced video clips into a virtual patient resource, replacing existing text content, and tested it with undergraduate learners as part of an interactive online PBL session. We explored the impact that this intervention had on participants’ ability to access and evaluate the information provided in the resource and their perceptions of how effective the approach was. The results identified both positive and negative effects from the introduction of the intervention. Students identified value in the video resource, but when asked to state a preference, the majority chose the text-based resource. Course of study (graduate entry or undergraduate entry), and accordingly varying levels of experience, did not impact learners’ stated preference.

Our results lead us to conclude that a combination approach may be a superior one within the context of undergraduate PBL. Using video only for elements to which it is particularly suited (ie, displaying procedures) may reduce any negative impact on the pace of the learning activity and would reduce any extraneous cognitive load introduced by video that might reduce the efficacy of the learning resource. Further research is necessary, in particular larger scale studies using a greater number of virtual patient interventions and contexts. However, despite the provisional nature of our findings, we have illustrated the context dependency of the perceived value of different multimedia components in a small-group PBL setting and in doing so we have developed a richer understanding of the role of educational multimedia in health professional education.

## Appendix

Multimedia Appendix 1 Video clip entitled “Surgical Progress”.

Multimedia Appendix 2 Video clip entitled “Packed red blood cells”.

Multimedia Appendix 3 Survey instrument for student participants.

Multimedia Appendix 4 Survey instrument for tutor participants.

Multimedia Appendix 5 Questions used to structure the tutor review meeting.

## Abbreviations

PBL: problem-based learning
