# Supplementary material for: Medical Student and Tutor Perceptions of Video Versus Text in an Interactive Online Virtual Patient for Problem-Based Learning: A Pilot Study
Source: J Med Internet Res. 2015 Jun 18;17(6):e151. doi: 10.2196/jmir.3922 (PMC4526950; doi:10.2196/jmir.3922)
Supplement: Multimedia Appendix 3 [file jmir_v17i6e151_app3.pdf]

This survey relates to your experience over the two tutorials completed this week, and asks you to compare your experiences of using text-based and video-based information in the tutorials. Please take the time to give us feedback on how you found these sessions. All responses are treated anonymously, and your opinions are extremely valuable to us.

Many thanks for your time.

1. Please select the room in which your PBL takes place.

- H1.1
- H1.2
- H1.5
- H1.6
- H1.7
- H4.3
- H4.4
- H4.8
- H4.9
- H4.11
- H4.12
- H4.13
- H4.14
- H4.15
- H4.16
- H4.19
- H4.20
- H4.21
- H4.22

2. Which course are you on?

- 4 year MBBS
- 5 year MBBS

3. On average, how many times did your group watch each video in Tutorial 2 before moving on in the case?

- Once
- Twice
- More than twice

Questions 4 and 5 require you to provide separate answers for both the text-based Tutorial 1, and the video-based parts of Tutorial 2. Please read each statement and select the option that most accurately describes your response for each type of tutorial.

4. While working on this case, I felt I had to make the same decisions a doctor would in real life.

	Strongly disagree	Disagree	Neutral	Agree	Strongly agree
Text-based tutorial	○	○	○	○	○
Video-based tutorial	○	○	○	○	○

5. While working on this case, I felt I were the doctor caring for this patient.

	Strongly disagree	Disagree	Neutral	Agree	Strongly agree
Text-based tutorial	○	○	○	○	○
Video-based tutorial	○	○	○	○	○

Please read the following statements and select the option that best describes your response.

6. Watching the scenario take place in the videos made me feel more emotionally involved with the case than when playing the role of an F2 doctor in the text.

Strongly disagree	Disagree	Neutral	Agree	Strongly agree
◯	◯	◯	◯	◯

7. Playing the role of an F2 doctor in the text-based parts of the tutorials increased my engagement with the scenario compared with watching the videos.

Strongly disagree	Disagree	Neutral	Agree	Strongly agree
◯	◯	◯	◯	◯

8. The use of video brought the scenario to life.

Strongly disagree	Disagree	Neutral	Agree	Strongly agree
◯	◯	◯	◯	◯

9. The use of video made the scenario more memorable.

Strongly disagree	Disagree	Neutral	Agree	Strongly agree
◯	◯	◯	◯	◯

10. The use of video influenced the option choices that my group made.

Strongly disagree	Disagree	Neutral	Agree	Strongly agree
◯	◯	◯	◯	◯

Please explain your answer

11. The use of video helped me to relate the scenario to real-life experience.

Strongly disagree	Disagree	Neutral	Agree	Strongly agree
◯	◯	◯	◯	◯

12. I was able to obtain all the information from the videos that I needed in order to make informed patient-management decisions.

Strongly disagree	Disagree	Neutral	Agree	Strongly agree
◯	◯	◯	◯	◯

13. I felt that it was easier to identify relevant information from text than the videos.

Strongly disagree	Disagree	Neutral	Agree	Strongly agree
◯	◯	◯	◯	◯

14. The use of video had a positive impact upon the group discussion.

Strongly disagree	Disagree	Neutral	Agree	Strongly agree
○	○	○	○	○

Please explain your answer

15. Do you feel that the use of video in the tutorial was effective?

- ○ Yes
- ○ No

Please explain your answer

16. Which form of scenario do you prefer?

- ○ Video-based
- ○ Text-based

Please explain your answer

17. Do you have any other comments?

Done
