# Supplementary material for: Medical Student and Tutor Perceptions of Video Versus Text in an Interactive Online Virtual Patient for Problem-Based Learning: A Pilot Study
Source: J Med Internet Res. 2015 Jun 18;17(6):e151. doi: 10.2196/jmir.3922 (PMC4526950; doi:10.2196/jmir.3922)
Supplement: Multimedia Appendix 4 [file jmir_v17i6e151_app4.pdf]

This survey relates to your experience over the two tutorials completed this week, and asks you to compare your experiences of tutoring using text-based and video-based information in the tutorials. Please take the time to give us feedback on how you found these sessions. All responses are treated anonymously, and your opinions are extremely valuable to us.

Many thanks for your time.

1. Please select the room in which your PBL takes place.

○ H1.1	○ H4.8	○ H4.16
○ H1.2	○ H4.9	○ H4.19
○ H1.5	○ H4.11	○ H4.20
○ H1.6	○ H4.12	○ H4.21
○ H1.7	○ H4.13	○ H4.22
○ H4.3	○ H4.14	
○ H4.4	○ H4.15	

2. On average, how many times did your group watch each video in Tutorial 2 before moving on in the case?

- ○ Once
- ○ Twice
- ○ More than twice

Please read the following statements and select the option that best describes your response.

3. The use of video influenced the decisions that my group made at option points.

Strongly disagree	Disagree	Neutral	Agree	Strongly agree
○	○	○	○	○

Please explain your answer

4. The use of video had a positive impact upon the group discussion.

Strongly disagree	Disagree	Neutral	Agree	Strongly agree
○	○	○	○	○

Please explain your answer

5. In this academic year, Stream B used an entirely text-based version of Tutorial 2, while Stream A used the partly video-based tutorial. If you have tutored both streams this year, did you notice any differences between the group dynamics of the two streams?

- ○ Yes
- ○ No
- ○ No difference
- ○ N/A, I didn't tutor both streams

Please explain your answer

6. Do you feel that the use of video in the tutorial was effective?

- ○ Yes
- ○ No

Please explain your answer

7. Which form of scenario do you feel works best for PBL?

- ○ Video-based
- ○ Text-based

Please explain your answer

8. Do you have any other comments?

Done
