# Supplementary material for: Medical Student and Tutor Perceptions of Video Versus Text in an Interactive Online Virtual Patient for Problem-Based Learning: A Pilot Study
Source: J Med Internet Res. 2015 Jun 18;17(6):e151. doi: 10.2196/jmir.3922 (PMC4526950; doi:10.2196/jmir.3922)
Supplement: Multimedia Appendix 5 [file jmir_v17i6e151_app5.pdf]

Tutor Review Session – Video vs Text

Introductory information

Thank you for participating in this discussion, and for tutoring the Albert Smith case. This discussion is aiming to capture your perceptions and experiences of tutoring the case and how you found that students responded to it. Your responses will be used as part of a research effort aiming at understanding online interactive-PBL sessions and how they can be designed to maximise their effectiveness and the learning experiences for students. We will be recording the discussion to allow us to make sure that we capture all the responses accurately. The results of the research will be potentially used for publication, but all comments will be anonymous and names will not be used anywhere in the research.

Questions

- How many people here were involved with tutoring the session with the videos?
- How many tutored the videos and the text version back in August?
- Briefly, what did you feel worked well with the videos?
- Briefly, what didn't work so well?
- How did the use of video impact upon your session?
    - In a text-based VP, students "are" the doctor, whereas in a video VP they "are watching" the doctor. Do you feel that the students were aware of this distinction, and do you think this changed the nature of the discussion?
    - When watching a video, students have to interpret the video and assess its content to identify relevant information to the scenario, whereas with text the relevant information may be more clearly identifiable.
        - Do you feel students struggled to identify the relevant information?
        - How many times did you have to watch the videos?
        - Do you feel that this helped or hindered the PBL process?
- Did the video affect the dynamic of the group compared with text-based?
    - Were students more or less engaged with the process?
        - Why do you think that was?
- How would you implement the video differently?
